# Off-label use of biologics and janus kinase (JAK) inhibitors for scarring alopecias: a narrative review

**DOI:** 10.1007/s00403-025-04301-z

**Published:** 2025-06-09

**Authors:** Priya Agarwal, Oghenevoke Ajuchi, Tess M. Lukowiak, Babar K. Rao

**Affiliations:** 1 Department of Dermatology, Rutgers Robert Wood Johnson Medical School, 1 Worlds Fair Drive, Somerset, NJ 08873 USA; 2 Department of Dermatology, Weill Cornell Medicine, New York, NY 10021 USA

**Keywords:** Scarring alopecia, Biologics, JAK inhibitors, Lichen planopilaris, Frontal fibrosing alopecia, Folliculitis decalvans, Central centrifugal cicatricial alopecia, Dissecting cellulitis, Discoid lupus erythematosus, TNF-α inhibitors, IL-17 inhibitors, IL-23 inhibitors, IFNAR1 inhibitors

## Abstract

Scarring alopecias, including lichen planopilaris, frontal fibrosing alopecia, folliculitis decalvans, central centrifugal cicatricial alopecia, discoid lupus erythematosus, and dissecting cellulitis cause permanent destruction of hair follicles, resulting in patches of hair loss that can be devastating for patients. Treatment options for scarring alopecias focus on disease stabilization and currently include corticosteroids and immunosuppressive agents, which often offer inconsistent disease improvements with waning patient satisfaction, especially in severe stages of the condition. Recent advances in therapeutics such as biologics and JAK inhibitors may offer some potential for disease stabilization and resolution through modulation of the inflammatory and immune-mediated pathways of scarring alopecias. This review examines literature reporting the off-label use of biologics and JAK inhibitors for the treatment of scarring alopecias. We find that TNF-α, IL-17, and JAK inhibitors demonstrate the most potential of currently available agents, with IL-23 and Interferon Alpha Receptor 1 inhibitors also showing some benefit.

## Introduction

Scarring alopecia, also known as cicatricial alopecia, refers to inflammatory hair loss conditions associated with permanent destruction of hair follicles and patches of hair loss [1]. Scarring alopecias carry a significant mental health burden for patients, leading to impaired quality of life and psychological distress [2]. Associated psychiatric comorbidities include anxiety, depression, and insomnia, which may impact treatment selection [3]. The most common forms of scarring alopecia are lichen planopilaris (LPP), frontal fibrosing alopecia (FFA), folliculitis decalvans (FD), dissecting cellulitis (DC), discoid lupus erythematosus (DLE), and central centrifugal cicatricial alopecia (CCCA).

LPP is a rare autoimmune disorder that causes inflammation and scarring of hair follicles, leading to progressive and permanent hair loss [4]. The exact etiology of LPP is unknown but it is hypothesized that T-lymphocytes mistakenly attack follicular antigens, resulting in selective destruction of hair follicles [4]. FFA is considered a variant of LPP that primarily affects the frontal and temporoparietal hairline [5]. It is a slow-progressing, lymphocytic scarring alopecia disorder which affects the bulge area of the hair follicle that contains stem cells vital for hair regeneration and growth [6].

FD is a rare condition characterized by neutrophil predominant inflammation of the hair follicles [7]. Although the etiology is unknown, FD is often associated with *Staphylococcus aureus*, which is thought to recruit neutrophils and cause inflammation [7]. DC is a chronic inflammatory condition of the scalp characterized by nodules, interconnecting sinus tracts, and alopecic patches [8]. The condition is believed to begin with follicular obstruction by keratinous materials within the hair follicles which eventually rupture, initiating an inflammatory response [8].

CCCA is a type of primary scarring alopecia that most commonly occurs in women of African descent and may arise from several factors including genetic predisposition and ethnic hair practices [9]. In CCCA, the inner layer of the hair follicle peels off prematurely, causing inflammation and damage [9]. DLE is a chronic autoimmune skin condition that, when it affects the scalp, can cause permanent hair loss due to scarring and inflammation around the affected hair follicles [10].

Scarring alopecias cause variable degrees of follicular hair destruction. Current treatment for scarring alopecia includes topical and intralesional corticosteroids, oral or topical antibiotics, finasteride/dutasteride, hydroxychloroquine, and minoxidil [6, 11]. While these treatments are intended to reduce inflammation and slow disease progression, their effectiveness is inconsistent, and their safety raises concerns. Topical and intralesional corticosteroids, for instance, are associated with significant adverse effects including cutaneous atrophy, hypopigmentation, scarring, and abscess formation [11, 12]. Furthermore, oral finasteride use is associated with mood disturbance, gynecomastia, decreased libido, erectile dysfunction, and hydroxychloroquine can cause gastrointestinal distress, vision disturbances, and skin discoloration [11, 13]. Patients are often also treated with topical minoxidil, to avoid systemic side effects such as fluid retention, pulmonary hypertension, and reflex tachycardia, however in this form it can cause scalp dryness, burning, redness, and allergic contact dermatitis [11].

Biologics are injectable proteins that obstruct the action of cytokines involved in the regulation of immune responses and inflammation [14]. The JAK-STAT pathway is involved in signal transduction in biological processes like apoptosis, immune regulation, and cell differentiation [15]. Continuous activation of this pathway is linked to the progression of immune and inflammatory diseases like scarring alopecia [15]. Numerous studies have leveraged the immunosuppressive capabilities of biologics and JAK-inhibitors in the off-label treatment of scarring alopecia. This paper aims to review the efficacy of these new and emerging treatment modalities, to elucidate the potential of these drugs to reduce the disease burden of scarring alopecias for affected patients.

## Methods

A comprehensive literature review of PubMed (Medline) and Scopus was conducted on 11/17/2024 using the following search string: (“scarring alopecia” OR “lichen planopilaris” OR “frontal fibrosing alopecia” OR “folliculitis decalvans” OR “central centrifugal cicatricial alopecia” OR “dissecting cellulitis” OR “discoid lupus erythematosus”) AND (“biologics” OR “JAK inhibitors” OR “monoclonal antibodies” OR “immunotherapy”). Articles in full-text English and relevant to the off-label treatment of scarring alopecia were included. Animal studies and review articles were excluded. References of articles included were also screened. Additional relevant studies published after the search date but before April 2025 were also included if deemed pertinent to the scope of this review.

## Results

### TNF-α inhibitors

11 papers investigating the efficacy of TNF-α inhibitors for scarring alopecias were identified (Table 1). TNF-α inhibitors such as infliximab, adalimumab, and certolizumab were often employed off-label for FD and DC. Multiple case reports and one larger-scale study with 23 patients demonstrated disease stabilization or remission for patients with recalcitrant FD with adalimumab therapy [16–19]. Another case report featuring a patient with FD found rapid remission and no recurrence or side effects with infliximab infusion [20].

Similar results were noted in the treatment of DC. A case report found complete cessation of inflammatory lesions with adalimumab treatment at seven-month follow-up [21]. One case report even noted partial hair regrowth in a DC patient following adalimumab treatment for three months [22]. In another study featuring three patients, adalimumab resulted in the resolution of clinical symptoms at eight weeks, however, biopsy demonstrated the continued presence of subcutaneous sinus tracts [23]. One of the patients in the study was discontinued on adalimumab after achieving remission at four months, however, disease rapidly relapsed by four weeks post-cessation and had to be restarted [23]. A patient in another study was discontinued on adalimumab at 60 weeks due to secondary treatment failure and started on infliximab instead, which resulted in disease control at 16 weeks [24]. Certolizumab pegol demonstrated efficacy in a pregnant DC patient, who had significant improvement in lesions with no adverse events [25].

The literature also revealed a case report demonstrating hair regrowth following the use of adalimumab for FFA [26].

**Table 1:** Selected studies evaluating scarring alopecia outcomes following TNF-α inhibitor use

Author	Indication	Enrollment	Study Drug Regimen	Concurrent Therapies	Outcomes
Lobato-Berezo et al. [16]	FD	3	Adalimumab subcutaneous injection 160 mg at week 0, 80 mg at week 2, and 40 mg every 2 weeks	Not reported	At three-month follow-up, patient 1 had improvement with adalimumab but then experienced a relapse which required drug discontinuation Patient 2 had mild improvement at one-month follow-up Patient 3 had great improvement at four-month follow-up
Kreutzer et al. [17]	FD; LPP	3 (2 FD; 1 LPP)	Adalimumab 40 mg subcutaneously every two weeks	Not reported	At two-month follow-up, marked remission was observed in patient 1 (FD) At 12-week follow-up, disease stabilization was observed in patient 2 (FD) and 3 (LPP)
Iorizzo et al. [18]	FD	23	Adalimumab was administered as subcutaneous injections of 160 mg at week 0, 80 mg at week 2, and 80 mg every other week	None	Clinical improvement was evident in all the patients, starting from the first month, and was maintained during the treatment Two patients discontinued the treatment because of insufficient improvement No serious adverse events reported except for mild gastrointestinal symptoms in two patients
Alsantali et al. [19]	FD	1	Adalimumab 80 mg loading dose was administered at week 0, and 40 mg every other week	Not reported	At three-month follow-up, disease stabilization was achieved with alleviation of inflammatory pustules At 10-month follow-up, no new disease foci or inflammation appreciable No serious adverse events were reported
Mihaljevic et al. [20]	FD	1	Infliximab 5 mg/kg	Not reported	Rapid remission after three infusions At 12-month follow-up, there was no recurrence No serious adverse events reported
Martin-Garcia et al. [21]	DC	1	Adalimumab 80 mg on day 0, 40 mg on day 7, and 40 mg every other week thereafter	Not reported	At one-month follow-up, the patient reported a significant decrease in pain and swelling of the lesions At seven-month follow-up, the patient was completely clear of inflammatory lesions No serious adverse events reported
Takahashi et al. [22]	DC	1	Adalimumab 80 mg on day 0, followed by 40 mg every other week thereafter (increased from 40 to 80 mg every other week at 3 months)	Not reported	At one-month follow-up, pain and purulent secretion were improved At three-month follow-up, partial hair regrowth was observed
Navarini et al. [23]	DC	3	Adalimumab 80 mg subcutaneously, followed by a dose of 40 mg 1 week later, and an additional 40 mg every second week	None	Within eight weeks, clinical symptoms subsided for all patients At three-month follow-up, patients’ symptoms improved however biopsy demonstrated subcutaneous sinus tracts remained unchanged When treatment was stopped in patient 3 after four months of successful treatment, disease activity returned within four weeks, and adalimumab had to be restarted
Sanchez-Diaz et al. [24]	DC	2	Adalimumab 80 mg every two weeks and/or Infliximab 5 mg/kg every eight weeks	Patient 1: Dapsone 100 mg/day for 20 weeks and ertapenem 1 g/day for six weeks Patient 2: None	Patient 1 was discontinued on adalimumab after 60 weeks due to secondary failure and started on infliximab; at 16-week follow-up, disease control was observed At 12-week follow-up, patient 2 maintained disease stabilization on adalimumab without need for adjuvant therapies
Almuhanna et al. [25]	DC	1	Certolizumab pegol subcutaneous injections with a loading dose of 400 mg at weeks 0, 2, and 4, followed by 200 mg every other week	None	At four-month follow-up, there was a 70% improvement of lesions At eight-month follow-up, treatment response was sustained No serious adverse events reported
Alam et al. [26]	FFA	1	Adalimumab (160 mg week 1; 80 mg week 2; 40 mg weekly beginning at week 4)	Leflunomide 20 mg/day	At three-month follow up, there was a reduction in inflammation of LPP patches At six-month follow up, further improvement was noted with no reported adverse effects

### IL-17 inhibitors

Six studies utilizing IL-17 inhibitors such as secukinumab and ixekizumab, which block IL-17 A, and brodalumab, which blocks the IL-17 receptor, in the treatment of scarring alopecia were identified (Table 2).

Secukinumab demonstrated improvement in FD for one patient in as quickly as two months in one case report [27]. At four-month follow-up, significant improvements were observed, with inflammation, pustules, and lesions diminished in severity [27]. In a separate report, secukinumab again showed rapid improvement in drainage, pain, and nodule formation in just one month for a patient with DC whose disease had previously been refractory to multiple treatments including adalimumab [28].

For patients with LPP, the efficacy of secukinumab was evaluated in a randomized double-blind placebo-controlled phase II trial [29]. While the study did not meet its primary endpoint of achieving an Investigator’s Global Assessment (IGA) score ≤ 2 at week 16 for the overall cohort, a higher proportion of patients in the LPP group receiving secukinumab (37.5%) achieved this response compared to those on placebo (30.8%) [29].

In another study assessing the efficacy of adalimumab and ixekizumab in treating FD, the patient initially showed improvement three months after starting adalimumab, however, a severe relapse occurred, leading to discontinuation [16]. The patient was subsequently treated with an ixekizumab regimen which was also discontinued due to disease worsening [16].

Furthermore, in the treatment of LPP, a case report found complete resolution of inflammatory lesions within three months following brodalumab 210 mg [30]. Additionally, another report demonstrated improvement in both the Lichen Planopilaris-Activity Index (LPPAI) and the Severity of Alopecia Tool (SALT) score after 12 weeks of ixekizumab [31]. The patient continued to display clinical improvement with no reported side effects after 12 months of treatment [31].

**Table 2:** Selected studies evaluating scarring alopecia outcomes following IL-17 A and IL-17R inhibitor use

Author	Indication	Enrollment	Study Drug Regimen	Concurrent Therapies	Outcomes
Lobato-Berezo et al. [16]	FD	1	Ixekizumab 160 mg subcutaneous injections at week 0, 80 mg at weeks 2, 4, 6, 8, 10, 12, and 80 mg every 4 weeks from the 16 weeks	Not reported	At 16-week follow-up, there was worsening of disease with an increase in the number of pustules, crusts, and the size of the alopecic patch prompting discontinuation Treatment failure attributed to paradoxical FD reaction
Ismail et al. [27]	FD	1	Secukinumab, 300 mg subcutaneously at weeks 0, 1, 2, 3, and 4 followed by 300 mg every 4 weeks thereafter	Ciclosporin 100 mg/day (discontinued after two months of secukinumab), minocycline 50 mg/day, topical clobetasol dipropionate 0.05%, and antiseptic shampoo	At two-month follow-up, mild clinical improvement noted At four-month follow-up, there was significant improvement with reduction in active disease No adverse effects reported
De Bedout et al. [28]	DC	1	Secukinumab, 150 mg subcutaneous monthly injections after 4 weekly loading doses	Dapsone 50 mg/day	At one-month follow-up, the patient’s drainage and pain ceased, with patient reporting regression of nodules for the first time in 6 years The patient developed an eczematous reaction after initiation of secukinumab which was treated topically and stabilized
Passeron et al. [29]	LPP	37	Secukinumab 300 mg every four weeks for 32 weeks or Placebo for 16 weeks followed by secukinumab 300 mg every 2 weeks for 16 weeks	None	Primary endpoint not met, however at week 16, IGA ≤ 2 for 37.5% of patients in secukinumab group vs. 30.8% in placebo group After switching from placebo to secukinumab every two weeks, IGA ≤ 2 for 63.6% of patients in placebo group
Maurelli et al. [30]	LPP	1	Brodalumab 210 mg every 2 weeks	Not reported	At three-month follow-up, there was complete remission of LPP lesions with disappearance of follicular hyperkeratosis and itch
Ahmed et al. [31]	LPP	1	Ixekizumab 160 mg initial dose administered subcutaneously, followed by 80 mg every 2 weeks for 12 weeks and a maintenance dose of 80 mg every 4 weeks	Topical clobetasol dipropionate alternating every two weeks with topical tacrolimus monohydrate 0.1% Discontinued at 20 weeks, and patient continued on ixekizumab monotherapy	At 12-week follow-up, the patient had a LPPAI score of 0 in comparison to an initial score of 6 (scale 1–10) as well as reduction in SALT score At 20 weeks, the patient had clinical improvement without relapse or side effects

### IL-23 inhibitors

Three studies assessing efficacy of IL-23 inhibitors for scarring alopecias were identified (Table 3).

In one study, risankizumab was found to alleviate existing nodules and abscesses in two patients with DC, while also preventing new lesions from forming [32]. One case report of tildrakizumab for DC demonstrated increased hair density and decreased number of pustules [33]. Furthermore, in the treatment of a patient with concomitant FFA and LPP refractory to multiple medications, tildrakizumab 100 mg resulted in sustained improvement in symptoms visualized clinically and via dermoscopy [34].

**Table 3:** Selected studies evaluating scarring alopecia outcomes following IL-23 inhibitor use

Author	Indication	Enrollment	Study Drug Regimen	Concurrent Therapies	Outcomes
Nagshabandi et al. [32]	DC	2	Risankizumab, in-clinic injection every 3 months	Patient 1: Not reported Patient 2: Topical clindamycin 1% solution daily	At 13-month follow-up, patient 1 reported improvement of roughly 70% in scalp lesions by the fifth dose, with no new lesions or draining nodules; signs of hair growth evident At four-month follow-up, patient 2 experienced improvements in skin lesions by the third dose
Awad et al. [33]	DC	1	Tildrakizumab, two doses of subcutaneous injection given 4 weeks apart	Not reported	At eight-week follow-up, the patient increased hair density and reduced the number of pustules Patient reported alleviated scalp tenderness and hair regrowth
Trindade de Carvalho et al. [34]	FFA/LPP	1	Tildrakizumab, 100 mg subcutaneously at weeks 0, 4, and subsequently 12 weekly	Minoxidil 1–2 mg/day and dutasteride 0.5 mg/day	At 16-week follow-up, patient experienced symptomatic improvements At 13-month follow-up, remission was maintained No adverse reactions reported

### JAK inhibitors

17 studies investigating the use of oral and topical JAK inhibitors in the treatment of scarring alopecia were identified (Table 4).

Three included studies looked at topical ruxolitinib 1.5% cream for FFA and LPP, the largest of which was a retrospective cohort study of 20 patients which found a mean reduction in LPPAI from 2.7 to 1.7 (*p* < 0.01) over a minimum of three months [35–37]. Another study featuring 41 patients using topical tofacitinib 2% cream found that patients had a 48% reduction in LPPAI scores at six months [38].

Oral tofacitinib, baricitinib, brepocitinib, and upadicitinib have also been investigated. A recent phase II pilot study evaluating oral tofacitinib for DLE featured two patients with scalp involvement who experienced 100% and 65% hair regrowth, respectively [39]. Oral tofacitinib has also been assessed for LPP, with eight out of 10 patients demonstrating clinical improvement and decreased LPPAI scores (*p* = 0.0014) [40]. Another LPP patient had similar results with oral tofacitinib, with evidence of clinical improvement and resolution of itch [41]. However, a case series that followed three patients with FD treated with oral tofacitinib found that despite initial improvement, all three patients suffered disease relapse of disease at one, six, and twelve months post drug cessation, respectively [42].

In case reports, baricitinib was shown to decrease disease progression in FFA and LPP [43–45]. A larger retrospective review of 12 patients with LPP or FFA treated with oral baricitinib showed an initial median LPPAI score reduction of 2.8 (46.5%; *p* = 0.043) in LPP and 5.6 (83.8%; *p* = 0.11) in FFA, however responses were sustained in only a subset after six months, with four patients failing to respond and two progressing despite dose escalation [45]. Oral baricitnib was also evaluated for four FD patients, all of who demonstrated a reduction in inflammation within one to three months [46]. One patient experienced relapse after self-discontinuation of the drug, but it was otherwise well tolerated [46]. A 44-year-old African American patient with CCCA demonstrated resolution of symptoms and partial hair regrowth in the medial parietal scalp after a two month trial of baricitinib 4 mg [47].

A recent phase 2a randomized, placebo-controlled trial with 50 patients evaluating brepocitinib as a therapy for LPP, FFA, and CCCA found that brepocitinib significantly reduced scalp expression of C-C motif chemokine ligand 5 (CCL5), a marker of IFNγ activity, and improved clinical severity scores, including LPPAI, Frontal Fibrosing Alopecia Severity Index (FFASI), and Central Hair Loss Grade (CHLG) across all disease subtypes by week 24, with further improvement through week 48 [48]. Subtype-specific analyses showed the most robust responses in LPP and FFA [48].

A case series of five patients with recalcitrant LPP treated with upadacitinib found that average LPPAI decreased from 8.7 to 1.3 (*p* = 0.043) [49]. Additionally, a case report demonstrated significantly fewer pustules, smaller sinus tracts, and overall decreased inflammation for a patient with recalcitrant DC treated with upadacitinib 15 mg twice daily [50].

**Table 4:** Selected studies evaluating scarring alopecia outcomes following JAK inhibitor use

Author	Indication	Enrollment	Study Drug Regimen	Concurrent Therapies	Outcomes
Desai et al. [35]	FFA	1	Topical ruxolitinib 1.5% cream once daily along the frontal hairline	Doxycycline hyclate 100 mg BID, hydroxychloroquine 200 mg BID, intralesional triamcinolone injections, tacrolimus 0.3%, topical clobetasol 0.05% solution, pioglitazone 15 mg/day, topical minoxidil 5% solution, oral minoxidil 5 mg/day, dutasteride 0.5 mg/day, and Excimer narrowband UV-B laser	At three-months, stabilization of the frontal hairline which was confirmed by clinical evaluation and trichoscopy No serious adverse events reported
Williams et al. [36]	LPP, FFA, LPP/FFA overlap	20 (6 LPP, 8 FFA, and 6 LPP/FFA overlap)	Topical ruxolitinib 1.5% cream twice a day (4/20, 20%), once a day (9/20, 45%), or every other day (7/20, 35%)	16/20 (80%) patients on concurrent therapies	Reduction in LPPAI scores from a mean of 2.7 to 1.7 (*p* < 0.01) The average reduction in LPPAI score was 34% Patients who applied topical ruxolitinib more frequently had a trend toward greater reductions in LPPAI scores (*p* = 0.06) Two reports of scalp irritation, one led to ruxolitinib discontinuation
Dunn et al. [37]	FFA	3	Topical ruxolitinib 1.5% BID (patients 1 and 2) Oral baricitinib 4 mg/day (patient 3)	Patient 1: Dutasteride 0.5 mg/day and minoxidil 1.25 mg/day Patient 2: Dutasteride 0.5 mg/day and minoxidil 1.25 mg/day (discontinued after six months of topical ruxolitinib) Patient 3: Dutasteride 0.5 mg/day, minoxidil 1.25 mg/day, doxycycline 100 mg/day, intralesional triamcinolone (5 mg/mL) every four weeks.	At 12-week and 15-week follow up, respectively, for the patients on topical ruxolitnib, there was reduction of itch, reduction in LPPAI and FFASI, and improvement in perifollicular erythema and scale sustained at six months Patient on oral baricitnib had complete resolution of perifollicular scale/erythema, symptomatic improvement in pruritus, and reduction in LPPAI and FFASI; Baricitinib was discontinued after two months of use with no recurrence noted at one month follow-up No serious adverse events reported
Chen et al. [38]	LPP; FFA	41 (3 LPP; 31 FFA; 7 LPP/FFA overlap)	Topical tofacitinib 2% cream	Used as monotherapy (*n* = 10, 24.4%) or adjunctive therapy (*n* = 31, 75.6%) with mean treatment duration of nine months	Patients demonstrated a significant reduction in LPPAI scores after receiving topical tofacitinib (*p* < 0.0001) with an overall 48% decrease in LPPAI score at 6 months Of FFA or FFA/LPP overlap patients, 92.1% exhibited improvement or stabilization of frontotemporal hairline, and 7.9% had progression of hairline recession Reported adverse events included scalp irritation in two patients (4.9%) and acne in one patient (2.4%)
Alsukait et al. [39]	DLE	5 (3 with scalp involvement)	Oral tofacitinib 5 mg BID	Two out of three patients with scalp involvement completed the study Patient 1: Hydroxychloroquine 400 mg/day Patient 2: None	Two out of three patients with scalp involvement completed the 24-week study and experienced 100% and 65% hair regrowth, respectively No serious adverse events reported
Yang et al. [40]	LPP	10	Oral tofacitinib 5 mg twice or three times daily for 2–19 months	Concurrent therapies used in five patients (50%)	Eight patients had clinical improvement with tofacitinib as either monotherapy (4/10) or adjunctive therapy (4/10) LPPAI before and after treatment was measured in seven patients decreased after treatment (*p* = 0.0014) One patient complained of 10 pound weight gain after 12 months on tofacitinib, with no other adverse events reported
Batra et al. [41]	LPP	1	Oral tofacitinib 5 mg BID	Dapsone	Clinical improvement on the crown and vertex with reduced scalp visibility and elimination of itch No serious adverse events reported
Jerjen et al. [42]	FD	3	Oral tofacitinib 2.5–5 mg/day	Patient 1: Minoxidil, ciclosporin (for eight months), clarithromycin, clindamycin shampoo, clobetasol propionate 0.05% Patient 2: Rifampicin (for two months), minoxidil, clarithromycin, clindamycin shampoo (for one month) Patient 3: Minoxidil, spironolactone, clarithromycin, clindamycin shampoo, clobetasol propionate 0.05%	At nine-month follow-up. patient 1 reported 80% itch improvement and full pain resolution, but relapsed one month after stopping treatment At three month follow up, patient 2 showed resolved inflammation; after stopping the drug at sixteen months, relapse occurred six months later At one month follow-up, patient 3 had cleared pustules and reduced hair shedding; relapse occurred twelve months after stopping at ten months Adverse events included liver function test abnormalities and indigestion (*n* = 1), and eosinophilia, hypercholesterolemia, and mild fatigue (*n* = 1)
Kreuter et al. [43]	FFA	1	Oral baricitinib 4 mg/day for two months followed by ongoing maintenance therapy of 2 mg/day	Not reported	Progression of hair loss was halted with resolution of erythema No serious adverse events reported
Li et al. [44]	LPP	1	Oral baricitinib 4 mg/day reduced to 2 mg/day at three months	None	At three-month follow up, erythema was reduced by 70% and hair loss stopped progressing At nine-month follow up, scalp erythema almost completely resolved No serious adverse events reported
Moussa et al. [45]	LPP; FFA	12 (7 LPP, 5 FFA)	Oral baricitinib 3.4–6.8 mg/day	All patients were on concurrent therapies	5/7 patients with LPP demonstrated an initial reduction in LPPAI (46.5%, *p* = 0.043); however, the response was maintained in only three patients after six months 3/5 patients with FFA demonstrated an initial reduction in LPPAI (83.8%; *p* = 0.11), with the response maintained in two patients after six months Two patients with LPP and two with FFA failed to improve despite baricitinib dose escalation and of these, two patients experienced disease progression Adverse events included transaminitis (*n* = 1), hypercholesterolemia (*n* = 1), neutropenia (*n* = 1), and fatigue (*n* = 1)
Moussa et al. [46]	FD	4	Oral baricitinib 3.4–6.8 mg/day	Patient 1: Clobetasol propionate 0.05%, oral minoxidil, topical clindamycin 1% Patient 2: Finasteride, intermittent intralesional triamcinolone, oral minoxidil, rifampicin Patient 3: Clindamycin shampoo 1%, oral minoxidil, mometasone furoate 0.1% Patient 4: Clindamycin shampoo 1%, doxycycline, intermittent intralesional triamcinolone, oral minoxidil	3/4 patients had reduction in inflammation and symptoms at 5–15 month follow up One patient relapsed two months after self-discontinuing drug Adverse events included hypercholesterolemia (*n* = 1) and transient acne (*n* = 1)
Workman et al. [47]	CCCA	1	Baricitinib 4 mg/day	Monthly intralesional triamcinolone injections (5 mg/mL)	At 1 month, symptoms were resolved At 2 months, clinical improvement in the medial parietal scalp was noted No serious adverse events reported
David et al. [48]	FFA, LPP, and CCCA	50 (26 FFA/LPP; 24 CCCA)	Brepocitinib 45 mg daily or placebo for 24 weeks, after which all patients received brepocitinib for another 24 weeks	None	CCL5 expression was significantly reduced in brepocitinib-treated patients at week 24 compared to baseline (*p* = 0.004) and placebo (*p* = 0.01) LPP patients had a 51.0% reduction in LPPAI at week 24 (*p* < 0.001) and 79.2% at week 48 (*p* < 0.001); FFA patients showed 33.6% and 39.0% reductions in FFASI at weeks 24 and 48 (*p* = 0.02, *p* < 0.01); CCCA patients had a 43.5% reduction in CHLG at week 48 (*p* < 0.001) Two serious adverse events (anemia; pneumonia with gastroenteritis) occurred in the brepocitinib group, leading to discontinuation
Lasheras-Perez et al. [49]	LPP	5	Upadacitinib 15 mg (1/5) and 30 mg (4/5)	Patient 1: Intralesional corticosteroids and oral minoxidil Patient 2: None Patient 3: None Patient 4: None Patient 5: Intralesional corticosteroids and oral minoxidil	Average LPPAI decreased from 8.7 to 1.3 (*p* = 0.043) Two patients developed mild acne and one patient had transient elevation of liver enzymes; no patient was discontinued on upadacitinib during follow up
Islam et al. [50]	DC	1	Upadacitinib 15 mg BID	Benzoyl peroxide 10%, sulfamethoxazole-trimethoprim 800 − 160 mg BID, and intralesional triamcinolone injections (40 mg/ml)	At a one-month follow-up, the patient reported substantial improvement in pain, pustular draining, and bleeding At two-month follow-up, exam revealed significantly fewer pustules, smaller sinus tracts, and overall decreased inflammation with no visible drainage No serious adverse events reported
Plante et al. [51]	LPP; FFA	9 (7 LPP, 2 FFA)	Topical tofacitinib 2% BID (*n* = 3) Oral tofacitinib 10–15 mg/day (*n* = 6)	All patients were on concurrent therapies	3/4 patients receiving topical therapy achieved a positive initial response, of which two patients exhibited sustained clinical improvement All patients receiving systemic therapy demonstrated a favorable initial response, and this was maintained in 5/6 patients Adverse events included mild transient hemoglobin and creatinine abnormalities (*n* = 1) and mild elevated triglycerides and hypercholesterolemia (*n* = 2)

### IFNAR1 inhibitors

We identified two case reports assessing anifrolumab, an interferon alpha receptor 1 inhibitor, for the treatment of DLE [51, 52]. One case report described a 27-year-old woman with scarring alopecia from DLE unresponsive to topical therapies or oral glucocorticoids [51]. However, one year after starting anifrolumab, she showed notable improvements in hair density and length, along with resolution of erythema [51]. In another case report, a 59-year-old woman with DLE was treated with anifrolumab after multiple therapies were ineffective [52]. She had improvement in cutaneous inflammation and acute hair loss—reflected in decreased Cutaneous Lupus Erythematosus Disease Area and Severity Index (CLASI)–Activity and Systemic Lupus Erythematosus Disease Activity Index 2000 (SLEDAI-2 K) scores—though notably there was no significant change in the CLASI–Damage score, which may indicate no significant change in scarring alopecia during the four weeks of the study [52].

## Discussion

Current treatment options for scarring alopecias generally include intralesional corticosteroids like triamcinolone, topical and oral corticosteroids, hydroxychloroquine, mycophenolate mofetil, oral minoxidil, anti-androgens such as finasteride and dutasteride, and other anti-inflammatory agents [53]. These treatments aim to slow disease progression, however, their efficacy varies among patients, and complete disease remission is often unattainable [53]. Furthermore, the long-term use of these treatments can be associated with significant adverse effects, including immunosuppression, lab abnormalities, hormonal changes, weight gain, and metabolic disturbances [54]. These challenges underscore the need for more tolerable therapies that address the underlying pathophysiology of scarring alopecia. Biologics and JAK inhibitors may have the potential to address this need, offering a more precise approach by modulating specific pathways involved in scarring alopecias and, in some cases, a more favorable side effect profile.

This review highlights studies featuring the off-label use of biologics and JAK inhibitors for the treatment of scarring alopecias. Taken together, these publications demonstrate the potential ability of biologics and JAK inhibitors to reduce inflammation, stabilize disease progression, and in limited cases, even promote hair growth. These findings are promising for patients experiencing the devastating effects of scarring alopecia, particularly those who may have failed standard of care treatments.

We find that TNF-α, IL-17, and JAK inhibitors may demonstrate some efficacy in managing scarring alopecias, most notably DC and FD, with some evidence for LPP, FFA, and CCCA. TNF-α inhibitors, including adalimumab, infliximab, and certolizumab, show evidence of clinical response in FD, DC, FFA, and LPP patients, with minimal adverse effects reported. In a cohort of 23 FD patients, adalimumab resulted in clinical improvement for all patients beginning in the first month of treatment, with sustained response [18]. Additional studies reported notable improvement with TNF-α inhibitors, indicating that these agents have largely favorable safety profiles and may serve as effective therapies for refractory scarring alopecias [16, 17, 19–26].

IL-17 pathway inhibitors also show promise. While the proof-of-concept PRELUDE study, featuring 111 patients (37 with LPP) did not ultimately meet criteria for robust treatment efficacy, secukinumab did have modest efficacy and a favorable safety profile for LPP, with sustained and improved responses over time, particularly with intensified dosing [29]. Multiple case reports showed improvement for LPP, DC, and FD with secukinumab, ixekizumab, and brodalumab with fast results [27, 28, 30, 31]. Notably, for one patient, there was worsening of disease with ixekizumab leading to discontinuation [16]. This patient had also been discontinued on adalimumab due to relapse, and the authors attribute both of these failures to paradoxical FD reactions [16].

JAK inhibitors—including tofacitinib, ruxolitinib, baricitinib, upadacitinib, and brepocitinib—have shown encouraging efficacy in reducing inflammation, stabilizing disease progression, and improving clinical severity scores across various scarring alopecias [35–50, 55]. Both oral and topical formulations have been used, with topical agents favored in some studies for their improved safety profile and tolerability [35–38, 55]. While early responses are promising, particularly in LPP and FFA, relapses following drug discontinuation have been frequently observed [37, 42, 45]. Brepocitinib, currently under investigation in a phase 2a trial, has demonstrated some efficacy with favorable tolerability [48]. A recent review had similar findings, noting that the targeted mechanism of JAK inhibitors may allow them to serve as alternatives to corticosteroids and antimalarials [56].

Limitations to this review include small sample sizes and short follow-up periods in the articles, and few randomized controlled trials. Additionally, subjective scoring measures and provider variability may affect the reliability of reported outcomes. The absence of standardized criteria for assessing improvement, combined with the inherently fluctuating course of scarring alopecias, further complicates interpretation. Furthermore, the use of concurrent therapies may hinder our ability to attribute clinical successes to any single agent. Paradoxically, it is also worth noting that though biologics may combat the disease processes in scarring alopecias, increasing reports of drug-induced scarring alopecias with some of these same medications have also been reported [57, 58].

## Conclusion

Biologics, including TNF-α, IL-17, IL-23, IFNAR1 inhibitors, as well as JAK inhibitors, may represent a new frontier in the treatment of scarring alopecias refractory to the standard of care by modulating key inflammatory pathways. This reviewed examined the available literature reporting on the off-label use of biologics and JAK inhibitors for the treatment of scarring alopecias. We find that TNF-α, IL-17, and JAK inhibitors demonstrate the most potential of currently available agents, with IL-23 and Interferon Alpha Receptor 1 inhibitors also showing some benefit. Despite potential benefits, these therapies remain off-label, and current evidence is largely limited to small case series and individual reports. As such, well-designed, placebo-controlled clinical trials are essential to establish their safety and true therapeutic value.

## Data Availability

No datasets were generated or analysed during the current study.
